# Comparison of surgically induced astigmatism following PreserFlo MicroShunt implantation versus trabeculectomy in Japanese patients with glaucoma

**DOI:** 10.1007/s10384-026-01355-y

**Published:** 2026-04-06

**Authors:** Yukako Ishiyama, Shiroaki Shirato, Makoto Aihara, Rei Sakata

**Affiliations:** 1 Department of Ophthalmology, Graduate School of Medicine and Faculty of Medicine, The University of Tokyo Hospital, 7-3-1 Hongo, Bunkyo-ku, Tokyo, 113-8655 Japan; 2 Yotsuya Shirato Eye Clinic, Tokyo, Japan

**Keywords:** Intraocular pressure, PreserFlo MicroShunt, Surgically induced astigmatism, Trabeculectomy, Visual acuity

## Abstract

**Purpose:**

We compared the magnitude of surgically induced astigmatism (SIA) following PreserFlo MicroShunt (PMS) implantation versus conventional trabeculectomy (Trab), with both procedures being performed with mitomycin C (MMC).

**Study design:**

Retrospective cohort study

**Methods:**

We included 113 eyes of 113 patients who underwent standalone PMS surgery and 78 eyes of 78 patients who underwent standalone Trab, all with no previous history of glaucoma surgery. Corneal astigmatism was measured preoperatively and at 3 months postoperatively by use of an automated keratometer. The mean SIA (M-SIA) and centroid SIA (C-SIA) were calculated by use of a dedicated vector analysis tool. Preoperative and postoperative intraocular pressure (IOP) and visual acuity (VA) were compared by use of paired* t* tests. The Mann–Whitney U test was used to compare the M-SIA between the groups.

**Results:**

Postoperatively, the IOP decreased from 20.9 to 11.5 mm Hg in the PMS group and from 20.6 to 9.3 mm Hg in the Trab group (*P* <0.001 for both). The C-SIA demonstrated an astigmatic shift toward the site of tube insertion in the PMS group and toward the scleral flap location in the Trab group. The M-SIA was significantly lower in the eyes treated with PMS than in those treated with Trab in the superonasal quadrant, but not in the superotemporal site. The VA remained stable in the PMS group, whereas a significant decline was observed in the Trab group.

**Conclusions:**

The PMS is an effective surgical option for reducing IOP while minimizing SIA at 3 months postoperatively. By better preserving visual function, the PMS may contribute to improved vision and overall patient satisfaction with the surgical outcomes.

**Supplementary Information:**

The online version contains supplementary material available at 10.1007/s10384-026-01355-y.

## Introduction

The PreserFlo MicroShunt (PMS; Santen Pharmaceutical) is an 8.5-mm-long glaucoma filtration device composed of poly(styrene-block-isobutylene-block-styrene). It is implanted beneath the conjunctiva and Tenon capsule via an ab externo approach, following a relatively straightforward surgical technique [1]. The device facilitates the drainage of aqueous humor through its lumen, leading to the formation of a bleb in the fornix region. Since its introduction into clinical practice, numerous studies over the past decade have consistently demonstrated its efficacy and safety [2–16].

Although many patients report experiencing blurred vision following filtration surgery, this is often transient and primarily attributed to hypotony-associated retinal changes, such as hypotony maculopathy or choroidal detachment, which typically resolve as the intraocular pressure (IOP) stabilizes. Another important contributor to postoperative visual disturbance is surgically induced astigmatism (SIA) [17]. Unlike cataract surgery, controlling astigmatism while effectively reducing IOP after glaucoma filtration surgery remains a significant challenge. SIA may lead to an early postoperative reduction in visual acuity (VA) and, in some cases, may persist after surgery. In eyes undergoing trabeculectomy (Trab) with mitomycin C (MMC), SIA typically aligns with the orientation of the scleral flap and often presents as oblique astigmatism [18, 19]. This pattern is attributed to multiple factors, including slight sinking of the corneal edge at the ostomy site following scleral tissue block removal, scleral cauterization, and variations in the number and tightness of the scleral flap sutures [20–24]. Furthermore, studies have demonstrated a correlation between low postoperative IOP and the degree of induced regular astigmatism 1 month after Trab with iridectomy [24]; these findings suggest that the globe is more susceptible to deformation under hypotonic conditions, exacerbating astigmatic changes. In contrast, PMS implantation does not involve scleral block excision or scleral flap creation and is associated with a lower risk of hypotony than that with Trab. These factors may contribute to reduced structural disruption and a low incidence of SIA.

SIA can be evaluated via 2 principal methods: the arithmetic mean of SIA (M-SIA), which considers only the magnitude of astigmatism irrespective of its axis, and the centroid of SIA (C-SIA), which incorporates magnitude and directional components [25]. Ando and colleagues have reported that the C-SIA provides a more comprehensive assessment of the overall trend of SIA than does the M-SIA [18]. Although several studies have evaluated the C-SIA following filtration surgery [18, 19, 26], no large-scale clinical study has assessed the C-SIA after PMS implantation. A previous study compared the effects of PMS and Trab on postoperative corneal astigmatism [27], with astigmatic changes assessed by use of anterior segment optical coherence tomography and autorefraction; the findings indicated no significant change in astigmatism up to 5 months postoperatively in the PMS group, whereas the Trab group exhibited a significant increase in astigmatism, suggesting a lower astigmatic impact associated with PMS. However, the interpretation of these findings remains limited by the small size of the PMS group and the absence of a direct comparison of the M-SIA between the 2 procedures.

Although the primary objective of filtration surgery is to reduce IOP and slow glaucoma progression, the preservation of postoperative visual function remains equally important. Therefore, understanding the impact of SIA as a potential cause of postoperative VA reduction is essential. We evaluated and compared SIA outcomes following PMS implantation and Trab, with consideration of the device insertion site and scleral flap location in patients with primary open-angle glaucoma (POAG).

## Patients and methods

This retrospective study included patients diagnosed with POAG who had no previous history of glaucoma surgery. Written informed consent was obtained from all the patients. The institutional ethics committee approved the study protocol (registration ID: 2217 [The University of Tokyo Hospital] and R6-19 [Japan Medical Association Ethics Review Committee]). The study was performed in accordance with the Declaration of Helsinki. Standalone MMC-augmented PMS implantation was performed between October 2022 and April 2023, whereas initial MMC-augmented Trab was conducted between March 2015 and April 2020. Eyes with a history of corneal refractive surgery or glaucoma surgery, including minimally invasive glaucoma surgery, and those in whom essential examinations could not be reliably performed, were excluded.

The medical records of patients who attended follow-up examinations were reviewed at specific time points: preoperatively and 3 months postoperatively. Routine slit-lamp and fundus examinations were conducted at each visit. Intraocular pressure was measured during office hours by use of a calibrated Goldmann applanation tonometer (Haag-Streit Surgical). Keratometric values were obtained preoperatively (within 6 months before surgery) and at 3 months postoperatively by use of an automatic keratometer (Tonoreff-II; Nidek). Best-corrected VA was measured by refraction using a Landolt ring VA chart before surgery and at 3 months postoperatively. The decimal VA values were converted to logarithm of the minimum angle of resolution (logMAR) values in accordance with a previously published method [28].

### Surgical procedures for PMS

Following the administration of sub-Tenon anesthesia with 2% lidocaine, a 5- to 6-mm fornix-based conjunctival incision was created along the limbus, superotemporal or superonasal, depending on the surgeon’s preference. After diathermy coagulation of the scleral bed, MMC was applied. Next, the surgical site was thoroughly irrigated with balanced salt solution (BSS) to remove any residual MMC. A double-step knife was used to create a scleral tunnel, starting from the scleral bed and entering the anterior chamber approximately 3 mm posterior to the corneal limbus. Subsequently, the PMS tube was inserted through this tract, released, and secured in position. After aqueous humor flow from the distal end of the tube had been confirmed, conjunctival closure was performed by use of wing sutures with 10-0 nylon (Mani).

### Surgical procedures for Trab

Following the administration of sub-Tenon anesthesia, a 5- to 6-mm fornix-based conjunctival incision was made along the limbus on either the superotemporal or the superonasal side. This was followed by cauterization of the scleral bed and the creation of a single rectangular scleral flap measuring 3 × 3 mm. Next, MMC was applied and rinsed with BSS to remove any residual agent. A sclerocorneal block (0.5 mm vertically × 2.0 mm horizontally) was excised, which was followed by peripheral iridectomy. The scleral flap was secured with four 10-0 nylon sutures, and additional sutures were placed as needed to modulate aqueous humor flow. Conjunctival closure was completed by use of wing sutures with 10-0 nylon (Mani). Postoperatively, laser suture lysis or bleb needling was performed to enhance aqueous outflow if the IOP remained elevated.

### Postoperative medical treatment

Postoperatively, the patients received topical 0.1% betamethasone and moxifloxacin ophthalmic solution 4 times daily. Moxifloxacin was discontinued within 1 month of surgery, whereas betamethasone was continued for a minimum duration of 6 months.

### Statistical analyses

The primary outcome measures of our study were the 3-month postoperative M-SIA and C-SIA for the PMS and Trab groups. These were calculated by use of double-angle plots generated with a freely available tool on the American Society of Cataract and Refractive Surgery (ASCRS) website [25, 29]. The arithmetic M-SIA represents the average magnitude of astigmatism regardless of axis, whereas the C-SIA is a vector-based measure that accounts for magnitude and direction. Since the C-SIA is a vector quantity and not suitable for direct numerical comparison, group comparisons between the 2 surgical procedures were based on the M-SIA values. Eyes were categorized according to superonasal or superotemporal surgical site, and the M-SIA was compared across the 4 quadrants by use of the Mann–Whitney U test. The preoperative and 3-month postoperative IOP and VA were analyzed by use of paired* t* tests. We performed a multiple linear regression analysis using the 3-month SIA as the dependent variable, with surgical procedure (PMS or Trab) as the main effect and the 3-month IOP (IOP_3M) as a covariate. The model specification was as follows:

Regression equation: Y_i_ = β_0_ + β_1_ Surgery_i_ + β2 IOP_3Mi_ + ε_i_.

Dependent variable: Y = |SIA|_3M_.

Main effect: Surgery (PMS = 1, Trab = 0).

Covariate: IOP_3__M_.

Statistical analyses were performed with JMP Pro (version 17.0, SAS Institute) and SPSS (version 23.0, IBM). Probability values <0.05 were considered significant.

## Results

In total, 191 eyes were included (113 PMS and 78 Trab). Table 1 presents the baseline demographics of the study participants. At 3 months postoperatively, both groups demonstrated a significant reduction in IOP when compared with that at baseline (from 20.9 ± 8.0 to 11.5 ± 3.8 mm Hg for PMS and 20.6 ± 7.8 to 9.3 ± 3.9 mm Hg for Trab, *P* <0.001). Visual acuity was preserved in the PMS group, with a nonsignificant change from 0.18 ± 0.37 to 0.21 ±0.39 (*P*=0.11). In contrast, a significant decline in VA was observed in the Trab group, worsening from 0.10 ±0.35 to 0.18 ±0.39 (*P* =0.001). A responder analysis was performed to assess clinically meaningful VA decline (≥0.20 logMAR). The proportion of eyes meeting this criterion was generally lower in the PMS group than in the Trab group, particularly in the superonasal quadrants. Detailed quadrant-specific results are provided in Supplementary Table S1.

**Table 1:** Patient demographics

	PreserFlo MicroShunt	Trabeculectomy
Total number of eyes	113	78
Superotemporal of the right eye	30	23
Superonasal of the right eye	23	15
Superotemporal of the left eye	22	22
Superonasal of the left eye	38	18
Age, years	63.4 ± 14.4	55.0 ± 12.5
Sex, male/female (number)	59/54	40/38
Intraocular pressure, mm Hg	20.9 ± 8.0	20.6 ± 7.8
Visual acuity, logMAR	0.18 ± 0.37	0.10 ± 0.35

Data are shown as means ± standard deviations

To directly assess the relationship between SIA and VA, we performed additional analyses. Spearman correlation analyses showed no significant correlations between the SIA magnitude and change in VA (3-month logMAR minus preoperative logMAR) across quadrants in either group (all *P* >0.05; Supplementary Table S2). In addition, stratified analyses comparing oblique (30°–60° or 120°–150°) versus nonoblique astigmatism showed no significant differences in 3-month VA (logMAR) in either group (all *P* >0.05; Supplementary Table S3).

**Fig. 1:**
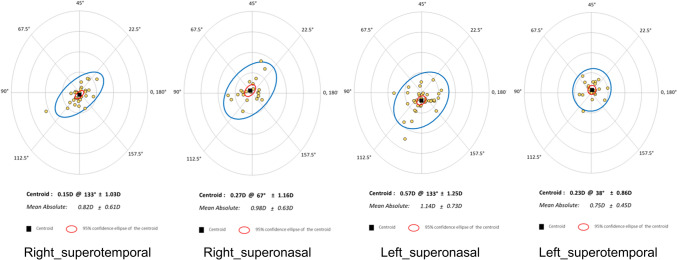
Centroid of surgically induced astigmatism (C-SIA) after PreserFlo MicroShunt implantation. C-SIA was calculated by use of double-angle vector plots generated with a freely available tool provided by the American Society of Cataract and Refractive Surgery. From left to right: superotemporal implantation of the PMS in the right eye, superonasal implantation in the right eye, superonasal implantation in the left eye, and superotemporal implantation in the left eye. The black square and yellow dots indicate the centroid of the SIA and the SIA of each eye, respectively. The red circle indicates the 95% confidence ellipse of the centroid, whilst the blue circle indicates the 95% confidence ellipse of the data set. Each ring indicates 0.5 D

**Fig. 2:**
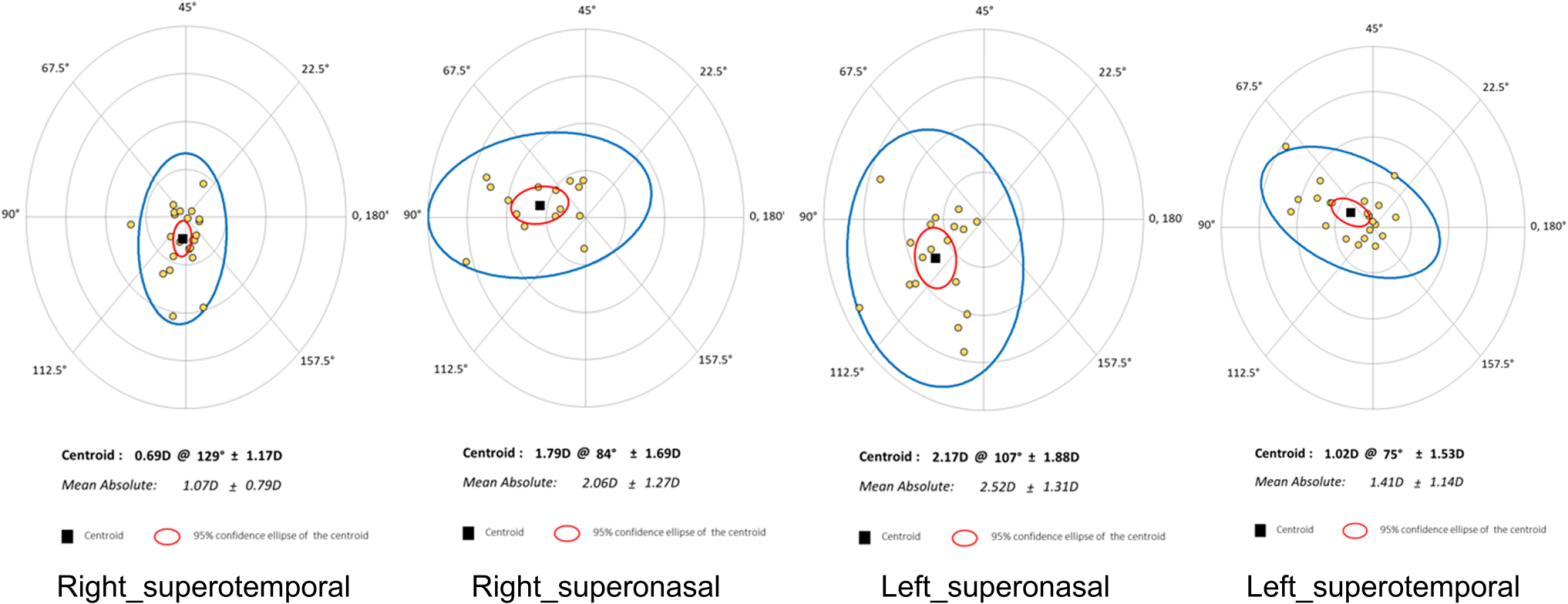
Centroid of surgically induced astigmatism (C-SIA) after trabeculectomy. C-SIA was calculated by use of double-angle vector plots generated with a freely available tool provided by the American Society of Cataract and Refractive Surgery. From left to right: superotemporal scleral flap formation in the right eye, superonasal flap in the right eye, superonasal flap in the left eye, and superotemporal flap in the left eye. The black square and yellow dots indicate the centroid of the SIA and the SIA of each eye, respectively. The red circle indicates the 95% confidence ellipse of the centroid, whilst the blue circle indicates the 95% confidence ellipse of the data set. Each ring indicates 0.5 D

The C-SIA, visualized using double-angle plots, is presented for the PMS group in Fig. 1. The C-SIA revealed a consistent astigmatic shift toward the PMS incision (ie, the microshunt insertion site) across all the quadrants. Similarly, the C-SIA after Trab is illustrated in Fig. 2, showing oblique astigmatism oriented in the direction of the scleral flap in all the quadrants, with a particularly pronounced effect in the superonasal quadrant. Overall, the magnitude of the C-SIA was greater in the Trab group than in the PMS group.

**Fig. 3:**
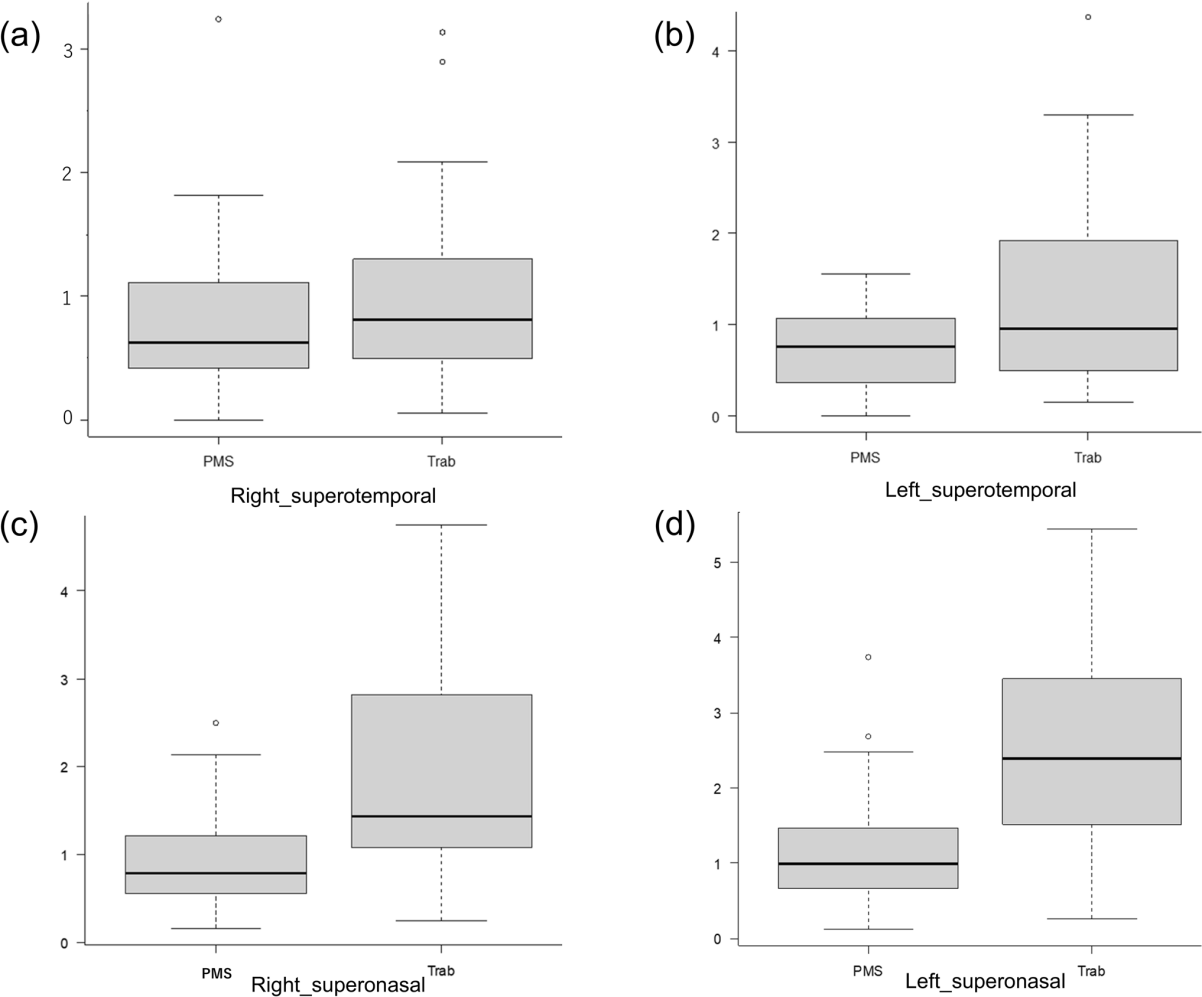
Mean surgically induced astigmatism in the superotemporal quadrant of the right eye. The vertical axis represents surgically induced astigmatism (in diopters), and the horizontal axis shows the 2 surgical groups (left: PreserFlo MicroShunt [PMS]; right: trabeculectomy [Trab]). The box plots display the interquartile range (IQR), with whiskers extending to 1.5 times the IQR from the first and third quartiles. The bars indicate the minimum and maximum values. **a**, **b**, **c**, and **d** indicate the superotemporal quadrant of the right eye, the superotemporal quadrant of the left eye, the superonasal quadrant of the right eye, and the superonasal quadrant of the left eye, respectively

At 3 months postoperatively, no significant differences in the M-SIA were observed between the PMS and Trab groups in the superotemporal quadrant of either eye. In the right eye, the M-SIA was 0.82 ± 0.61 D in the PMS group and 1.07 ± 0.79 D in the Trab group (*P* = 0.23; Fig. 3a). Similarly, in the left eye, the M-SIA measured 0.75 ± 0.45 D and 1.41 ± 1.14 D for the PMS and Trab groups, respectively (*P* = 0.08; Fig. 3b). In contrast, a significant difference was observed in the superonasal quadrant, where the M-SIA was significantly lower in the PMS group than in the Trab group. For the right eye, the M-SIA was 0.98 ± 0.63 D in the PMS group and 2.10 ±1.27 D in the Trab group (*P* < 0.001; Fig. 3c). In the left eye, the values were 1.14 ±0.73 D and 2.52 ± 1.31 D for the PMS and Trab groups, respectively (*P* <0.001; Fig. 3d).

After adjustment for postoperative IOP at 3 months, the surgical procedure remained a significant predictor of M-SIA in the superonasal quadrant in both eyes, whereas no significant association was observed in the superotemporal quadrant. The postoperative IOP itself was not a significant covariate in any quadrant (Supplementary Table S4).

## Discussion

SIA following glaucoma filtration surgery can adversely affect uncorrected and best-corrected VA. Consequently, even when the primary objective of surgery—namely, reduction of IOP to slow visual field progression—is achieved, patients may report diminished postoperative satisfaction owing to visual disturbances associated with astigmatic changes. Trabeculectomy, which involves scleral flap creation and suturing, frequently induces alterations in the corneal curvature, increasing the likelihood of postoperative astigmatism [30]. In contrast, minimally invasive procedures, such as PMS implantation, involve less disruption of the ocular structures and might be associated with a reduced risk of inducing SIA. Consequently, the extent of SIA has become an important consideration in surgical decision-making. Tolerance to astigmatic changes varies among patients and is influenced by several factors, including pre-existing corneal geometry, history of cataract surgery, and patient-specific visual demands and lifestyle. From a personalized medicine standpoint, evaluation of SIA is essential in guiding the choice of surgical technique and determining the optimal site of incision.

We analyzed postoperative astigmatism following initial PMS implantation and performed a comparative analysis with Trab, which served as the control group. Corneal astigmatism was measured with an autorefractor keratometer [18, 19]. Both surgical procedures resulted in a significant IOP reduction at the 3-month postoperative mark. C-SIA analysis demonstrated a pattern of oblique astigmatism in both groups, oriented in accordance with the PMS insertion site and with the scleral flap location in the Trab group. Furthermore, M-SIA analysis revealed that PMS implantation induced significantly less astigmatism than did Trab. These findings suggest that PMS implantation may have a reduced impact on corneal refractive status and better preserve postoperative VA when compared with conventional Trab. The responder-based approach provides a more clinically relevant perspective than do mean changes alone. It suggests that a subset of eyes—particularly after Trab in the superonasal quadrant—experienced meaningful VA decline at 3 months.

Regarding the magnitude of SIA following Trab, previous studies have reported M-SIA values ranging from 0.5 to 1.25 D [18, 19, 24, 26, 31–33]. Ando and colleagues analyzed 185 glaucomatous eyes undergoing Trab with MMC at a superonasal site and reported C-SIA values of 0.73 ± 1.02 D at 64° in the right eyes and 0.60 ± 1.03 D at 117° in the left eyes [18]. Their findings demonstrated astigmatic shifts oriented toward the scleral flap, with a consistent trend toward oblique astigmatism. Similarly, Shiratori and colleagues investigated 66 Japanese glaucoma eyes that underwent superotemporal Trab with MMC and reported a comparable oblique astigmatic shift corresponding to the flap location [19]. Their findings suggested that scleral flap suturing plays a pivotal role in inducing corneal steepening in the direction of the flap, whereas the choice of surgical technique—limbus-based versus fornix-based—had minimal effect on the extent of SIA.

In the present study, we found that, even after adjustment for IOP, the surgical procedure (PMS implantation vs Trab) had a significant impact on the M-SIA in the superonasal quadrant, whereas no such effect was observed in the superotemporal quadrant. One possible explanation for this asymmetry is the difference in anterior scleral morphology between the nasal and temporal sides. The superonasal sclera has been reported to have a larger radius of curvature and is therefore relatively flatter than the superotemporal sclera [34]. From a biomechanical standpoint, local traction and stress induced by scleral flap creation and suturing may be less efficiently dispersed in this flatter region. As a result, deformation around the flap may be more directly translated into changes in corneal shape, manifesting as surgically induced astigmatism. In contrast, the superotemporal sclera is slightly more convex, which may allow stress to dissipate 3-dimensionally, thereby reducing the impact of similar surgical manipulations on the cornea. Another hypothesis is that in the superonasal quadrant, the conjunctiva and Tenon capsule covering the scleral flap tend to have less redundancy and to be relatively tight. Thus, traction associated with bleb formation may be transmitted more directly to the limbal area, leading to peripheral corneal shape changes and, consequently, astigmatism. In contrast, the superotemporal quadrant generally has more “space” and tissue redundancy, allowing the conjunctiva and Tenon capsule to absorb part of the mechanical stress through tissue “slack,” which may attenuate the influence of bleb expansion on the corneal morphology. Taken together, these structural characteristics suggest that in the superonasal quadrant, mechanical forces are less likely to dissipate and more likely to be transmitted to the cornea, making the effect of the surgical procedure on the M-SIA more apparent. Conversely, in the superotemporal quadrant, the same surgical maneuvers may have a relatively smaller impact on corneal astigmatism.

Several hypotheses have been proposed to explain the occurrence of SIA following Trab. One proposed mechanism involves the excision of a 2- × 4-mm scleral-corneal block beneath the scleral flap, which may cause localized depression of the corneal margin at the surgical site, leading to steepening of the vertical corneal meridian and subsequent regular astigmatism [20]. In addition, scleral flap suturing can induce localized tissue compression, contributing to regular astigmatism [22]. Excessive intraoperative scleral cauterization and the number of scleral flap sutures have been implicated as potential contributors to SIA [23]. In contrast, the PMS procedure avoids scleral flap creation, suturing, and block excision, preserving the structural integrity of the globe. Consequently, PMS implantation is considered a surgical technique with less potential than Trab for inducing SIA .

In eyes undergoing Trab, a significant association has been reported between lower postoperative IOP at 1 month and the magnitude of SIA [24]. For instance, an IOP of 3 mm Hg was associated with approximately 1 D of regular astigmatism, whereas an IOP of 11 mm Hg had no significant effect on the corneal curvature. This correlation was diminished at 6 months postoperatively. It is plausible that during periods of low IOP, the globe is more susceptible to deformation, exacerbating astigmatic changes. The PMS procedure is less likely to induce postoperative hypotony than is Trab. This characteristic may contribute to the reduced magnitude of SIA observed with PMS because more stable IOP minimizes structural deformation of the globe.

Furthermore, the use of MMC has been implicated in the development and persistence of SIA. In eyes undergoing Trab without MMC, SIA stabilized within 3 months postoperatively, whereas in those treated with MMC, regular astigmatism persisted up to 12 months [21]. One possible mechanism is the inhibition of fibroblast proliferation by MMC, which alters the normal wound healing process and may prolong corneal remodeling. However, in the present study both PMS implantation and trabeculectomy were performed with MMC; therefore, the between-group differences in the SIA cannot be attributed to MMC use itself. Rather, it is likely that MMC amplifies the effects of the underlying surgical technique. In Trab, broad scleral flap creation, multiple flap sutures, and wide subconjunctival dissection expose a relatively large limbal area to MMC, which may lead to more extensive and asymmetric scleral and limbal remodeling and thereby sustain surgically induced changes in corneal curvature. By contrast, PMS implantation is performed through a relatively narrow intrascleral tunnel with more localized tissue dissection, so the area of MMC-modulated wound healing is smaller and more confined. This interaction between MMC and surgical technique may therefore explain why SIA was greater after Trab than after PMS in the present series.

To minimize SIA associated with Trab, it is essential to limit the size of the scleral-corneal block excision to the absolute minimum and to avoid excessive tension when securing the scleral flap sutures. Our findings revealed a significant difference in the magnitude of the M-SIA between the superonasal and superotemporal quadrants in eyes undergoing Trab. This discrepancy may be partly explained by variations in the ease of scleral flap suturing and conjunctival closure depending on the surgical quadrant. The superotemporal quadrant offers a wider surgical field, allowing for more controlled and precise suturing, which may reduce the extent of induced astigmatism. In contrast, the superonasal quadrant presents a more restricted operative field, leading to suboptimal wound closure. In addition, differences in tissue tension, wound healing dynamics, and the degree of conjunctival manipulation between the superonasal and superotemporal quadrants may contribute to the observed variation in the M-SIA. Further studies investigating quadrant-specific surgical techniques and their influence on SIA may yield valuable insights for refining Trab procedures and improving visual outcomes.

The Ex-PRESS shunt, which eliminates the need for scleral-corneal block excision, has been shown to induce less SIA than does Trab [32]. In addition, creating the filtration bleb closer to the fornix may help to reduce the impact of surgery on the corneal curvature and minimize SIA.

In this context, the PMS procedure, as a minimally invasive filtration surgery, may be particularly effective in limiting postoperative SIA. Unlike trabeculectomy, PMS implantation does not require the creation of a broad-based scleral flap; instead, a relatively narrow intrascleral tunnel is fashioned to accommodate the microshunt tube. This smaller and more localized scleral dissection is likely to reduce circumferential distortion of the limbal architecture and thereby attenuate surgically induced changes in corneal curvature. Nevertheless, the PMS procedure still involves conjunctival and Tenon-capsule suturing, topical application of MMC, and focal scleral cauterization, all of which can induce biomechanical alterations around the limbus and modulate wound healing. Tension in the conjunctiva and Tenon capsule overlying the device and bleb may transmit traction to the peripheral cornea, whilst MMC-related changes in tissue remodeling may prolong or enhance these effects. Taken together, these factors could explain why a vector shift of postoperative SIA toward the implantation site is observed even with PMS implantation, although its magnitude appears less pronounced than that seen after trabeculectomy in the present series.

Cataract formation is a known complication of filtration procedures; however, the reduced SIA associated with PMS implantation is expected to decrease the need for subsequent astigmatic correction. Moreover, when PMS implantation is performed in combination with cataract surgery, the likelihood of achieving the targeted intraocular lens power improves, as postoperative corneal alterations are minimized. Therefore, combined PMS implantation and cataract surgery may represent an advantageous approach for optimizing refractive and visual outcomes.

Our study has several limitations. First, the sample size was relatively small, as PMS continues to be adopted alongside minimally invasive glaucoma surgeries and traditional Trab, and therefore, the number of eligible cases remains limited. As PMS implantation becomes more widely implemented as the first-choice surgery, it will be possible to collect data on the incidence of astigmatism under various patient backgrounds and surgical conditions, leading to research involving a more diverse set of cases. Although the small sample size in the current study is acknowledged, it is anticipated that future studies, as PMS implantation continues to be adopted, will address this limitation. The expansion of PMS usage will enable larger-scale studies, thereby providing more robust evidence and contributing to a deeper understanding of its long-term impact.

Second, our analysis was based on overall corneal astigmatism and did not separately evaluate anterior and posterior corneal components. Ideally, detailed corneal shape analysis using anterior segment optical coherence tomography would yield more accurate and comprehensive data [35]. However, owing to the unavailability of this technology across all the participating centers, keratometric readings were used for analysis. Nonetheless, since posterior corneal astigmatism contributes significantly less to total astigmatism than does the anterior surface, its influence on the overall results is likely minimal.

Third, the timing of the postoperative assessments may have influenced the findings. The evaluation of corneal astigmatism at different postoperative intervals can yield varying results, and no consensus currently exists regarding the optimal time points for assessment. Previous studies have assessed astigmatic changes at 3 months, whereas others have extended follow-up to 12 months [18, 19, 24, 30]. Notably, several studies suggest a gradual shift toward regular astigmatism up to 12 months postoperatively in eyes undergoing Trab with MMC [36]. Thus, long-term follow-up is warranted to fully characterize the evolution of SIA over time. However, as the most significant decline in VA following filtration surgery typically occurs within the first 3 months, our focus on SIA during this early postoperative period remains justified. In our study, changes in VA during the early postoperative period (days to weeks after surgery) were not evaluated. Therefore, transient VA reduction related to SIA in the immediate postoperative phase cannot be determined from the present data. Future studies with more frequent and earlier postoperative visual assessments are warranted to clarify the temporal relationship between SIA and VA changes.

And, finally, there may be concerns regarding the potential differences in patient backgrounds between the 2 groups. However, since the primary focus of this study is on postoperative astigmatism (SIA), we believe that differences in preoperative background, such as IOP and corneal shape, are less likely to have significantly impacted the findings. Therefore, the lack of strict alignment of preoperative data between the 2 groups does not pose a significant issue in the context of our study.

In conclusion, our findings revealed that at 3 months postoperatively, the C-SIA associated with MMC-augmented PMS implantation exhibited steepening at the site of tube insertion, whereas the M-SIA was lower than that associated with MMC-augmented Trab. As a minimally invasive filtration procedure, PMS implantation offers a good IOP control while preserving the ocular structural integrity to a greater extent. Although the differences in SIA between these surgical techniques may diminish over time, our findings indicate that the PMS induces less astigmatic change at 3 months postoperatively. This reduction may contribute to improved postoperative quality of vision and overall quality of life. These findings offer valuable insights to guide surgical decision-making and support the consideration of the PMS as a favorable option for preserving visual function and enhancing patient quality of life following glaucoma surgery.

## Supplementary Information

Below is the link to the electronic supplementary material.

Supplementary file1 (DOCX 17 KB)

Supplementary file2 (DOCX 17 KB)

Supplementary file3 (DOCX 19 KB)

Supplementary file4 (DOCX 21 KB)
