# Supplementary material for: Comparison of surgically induced astigmatism following PreserFlo MicroShunt implantation versus trabeculectomy in Japanese patients with glaucoma
Source: Jpn J Ophthalmol. 2026 Apr 6;70(4):931–9. doi: 10.1007/s10384-026-01355-y (PMC13452854; doi:10.1007/s10384-026-01355-y)
Supplement: Supplementary file 1 [file 10384_2026_1355_MOESM1_ESM.docx]

**Supplementary Table S1** Responder analysis of clinically meaningful visual acuity decline (≥0.20 logMAR) at 3 months, by quadrant and procedure

| Quadrant | PreserFlo MicroShunt | Trabeculectomy |
| --- | --- | --- |
| Right–superonasal | 2 eyes (9%) | 2 eyes (13%) |
| Right–superotemporal | 4 eyes (13%) | 2 eyes (9%) |
| Left–superonasal | 5 eyes (13%) | 7 eyes (39%) |
| Left–superotemporal | 4 eyes (18%) | 5 eyes (22%) |

Responder definition: clinically meaningful visual acuity decline defined as ≥0.20 logMAR (≈2 ETDRS lines).
