# Supplementary material for: Comparison of surgically induced astigmatism following PreserFlo MicroShunt implantation versus trabeculectomy in Japanese patients with glaucoma
Source: Jpn J Ophthalmol. 2026 Apr 6;70(4):931–9. doi: 10.1007/s10384-026-01355-y (PMC13452854; doi:10.1007/s10384-026-01355-y)
Supplement: Supplementary file 2 [file 10384_2026_1355_MOESM2_ESM.docx]

**Supplementary Table S2** Spearman correlations between |SIA| and change in visual acuity (ΔlogMAR) at 3 months, by quadrant and procedure

|  | PMS | *P* value |
| --- | --- | --- |
| Right_superonasal | -0.14 | .51 |
| Right_superotemporal | -0.15 | .42 |
| Left_superonasal | -0.061 | .72 |
| Left_superotemporal | 0.092 | .68 |

*PMS* PreserFlo Microshunt, *Trab* trabeculectomy
