# Supplementary material for: Comparison of surgically induced astigmatism following PreserFlo MicroShunt implantation versus trabeculectomy in Japanese patients with glaucoma
Source: Jpn J Ophthalmol. 2026 Apr 6;70(4):931–9. doi: 10.1007/s10384-026-01355-y (PMC13452854; doi:10.1007/s10384-026-01355-y)
Supplement: Supplementary file 3 [file 10384_2026_1355_MOESM3_ESM.docx]

**Supplementary Table S3** Visual acuity change at 3 months stratified by oblique vs nonoblique SIA axis, by quadrant and procedure

|  | Oblique | | Nonoblique | |  |
| --- | --- | --- | --- | --- | --- |
| PMS | VA_3M | ±SD | VA_3M | ±SD | *P* value |
| Right_superonasal | 0.29 | 0.41 | 0.13 | 0.23 | .34 |
| Right_superotemporal | 0.10 | 0.33 | 0.21 | 0.35 | .51 |
| Left_superonasal | 0.02 | 0.24 | 0.27 | 0.47 | .14 |
| Left_superotemporal | 0.16 | 0.33 | 0.37 | 0.48 | .30 |

*PMS* PreserFlo Microshunt, *M* month, *VA* visual acuity, *SD* standard deviation

|  | Oblique | | Nonoblique | |  |
| --- | --- | --- | --- | --- | --- |
| Trab | VA_3M | ±SD | VA_3M | ±SD | *P* value |
| Right_superonasal | No data* | | 0.16 | 0.53 | NA |
| Right_superotemporal | 0.11 | 0.21 | 0.22 | 0.37 | .55 |
| Left_superonasal | -0.04 | 0.040 | 0.27 | 0.35 | .20 |
| Left_superotemporal | 0.41 | 0.59 | 0.16 | 0.31 | .35 |

*Trab* trabeculectomy, *VA* visual acuity, *M* month, *NA* not applicable, *SD* standard deviation

*No data indicates that there were zero eyes in the corresponding subgroup; therefore, mean/SD and *P* values could not be calculated (*P* value shown as NA).
