# Supplementary material for: Comparison of surgically induced astigmatism following PreserFlo MicroShunt implantation versus trabeculectomy in Japanese patients with glaucoma
Source: Jpn J Ophthalmol. 2026 Apr 6;70(4):931–9. doi: 10.1007/s10384-026-01355-y (PMC13452854; doi:10.1007/s10384-026-01355-y)
Supplement: Supplementary file 4 [file 10384_2026_1355_MOESM4_ESM.docx]

| Right_superonasal | | | | |  |  |
| --- | --- | --- | --- | --- | --- | --- |
|  | Coefficient | Standard error | *t* value | *P* value | Lower 95% | Upper 95% |
| Intercept | 2.45 | 0.39 | 6.27 | <.001 | 1.65 | 3.24 |
| Surgery（PMS) | -1.05 | 0.32 | -3.24 | .003 | -1.70 | -0.39 |
| IOP_3M_ | -0.039 | 0.031 | -1.28 | .21 | -0.10 | 0.023 |
|  |  |  |  |  |  |  |
| Right_superotemporal | | | | |  | |
|  | Coefficient | Standard error | *t* value | *P* value | Lower 95% | Upper 95% |
| Intercept | 0.85 | 0.33 | 2.56 | .014 | 0.18 | 1.51 |
| Surgery（PMS) | -0.31 | 0.22 | -1.41 | .16 | -0.75 | 0.132 |
| IOP_3M_ | 0.024 | 0.031 | 0.77 | .44 | -0.039 | 0.087 |
|  |  |  |  |  |  |  |
| Left_superonasal | | | | |  |  |
|  | Coefficient | Standard error | *t* value | *P* value | Lower 95% | Upper 95% |
| Intercept | 2.47 | 0.40 | 6.12 | <.001 | 1.66 | 3.28 |
| Surgery（PMS) | -1.38 | 0.29 | -4.74 | <.001 | -1.96 | -0.79 |
| IOP_3M_ | 0.0052 | 0.036 | 0.14 | .89 | -0.066 | 0.077 |
|  |  |  |  |  |  |  |
| Left_superotemporal | | | | |  | |
|  | Coefficient | Standard error | *t* value | *P* value | Lower 95% | Upper 95% |
| Intercept | 1.80 | 0.38 | 4.74 | <.001 | 1.03 | 2.57 |
| Surgery（PMS） | -0.57 | 0.29 | -1.98 | .055 | -1.16 | 0.012 |
| IOP_3M_ | -0.045 | 0.038 | -1.19 | .24 | -0.12 | 0.031 |

**Supplementary Table S4** Multiple linear regression of 3-month |SIA| with surgical procedure and 3-month IOP (IOP_3M) as covariate, by quadrant

*PMS* PreserFlo Microshunt, *IOP* intraocular pressure, *M* month
